# PVDF-based flexible piezoelectric tactile sensor for slip estimation using robotic gripper

**DOI:** 10.3389/frobt.2025.1691688

**Published:** 2025-12-10

**Authors:** Muhammad Hisyam Rosle, Abdul Rashid Saffiai, Abdul Nasir, Muhammad Nur Farhan Saniman

**Affiliations:** 1 Malaysia–Japan International Institute of Technology, Universiti Teknologi Malaysia, Kuala Lumpur, Malaysia; 2 Center for Artificial Intelligence and Robotics (CAIRO), Universiti Teknologi Malaysia, Kuala Lumpur, Malaysia; 3 Faculty of Electrical Engineering and Technology, Universiti Malaysia Perlis, Arau, Malaysia; 4 Faculty of Electrical and Electronic Engineering Technology, Universiti Malaysia Pahang Al-Sultan Abdullah, Pekan, Malaysia; 5 Mechanical Engineering Section, Universiti Kuala Lumpur, Malaysia France Institute, Bandar Baru Bangi, Malaysia

**Keywords:** piezoelectric, tactile sensor, slip estimation, robotic gripper, soft sensor

## Abstract

Robotic grippers are widely utilized in industrial manufacturing, but object slippage during assembly poses challenges, including potential damage, delays, and increased costs. Therefore, early slip detection is crucial for efficient manufacturing operations. Piezoelectric tactile sensors using polyvinylidene fluoride (PVDF) have been developed to detect vibrations. Nevertheless, the development of such sensors with a simple structure and lower fabrication cost, continues to be a challenging task. The analysis on the effect of the thicknesses of soft body layers that attached to sensing elements on the slip sensor’s performance has yet been discussed. In this project, a simple-structured and low-cost design of a flexible piezoelectric tactile sensor based on PVDF to estimate slip using robotic gripper is presented. The effect of different thicknesses of soft body layer made of silicone rubber and the sensor’s performance in detecting slip is discussed. A PVDF-based sensor is attached to soft body layer that is incorporated into a robotic gripper. Experimental results demonstrate that sensor sensitivity increases with lower soft body layer thickness. Additionally, the sensor’s signal amplitude increases with object load, indicating slip intensity. This advancement addresses challenges in fabricating simple structures and cost-effective piezoelectric sensors which enhance robotic gripper functionality in industrial applications.

## 1 Introduction

In recent years, there has been a growing interest in the utilization of soft and flexible materials in the development of robotic systems. Soft robotics, which exploit the mechanical compliance resulting from their composition of soft and flexible materials, possess the capability to effectively navigate uncertain and unstructured task environments (Shan et al., 2023; Babu et al., 2022). A broad spectrum of soft robotics applications has been discovered, such as soft wearable devices for medical purposes (Rosle et al., 2024; Pan et al., 2022), soft robotic grippers for agricultural and manufacturing product handling (Chu et al., 2023; Liu et al., 2023), and soft sensors for multipurpose sensing (Rosle et al., 2022; Rosle et al., 2020; Martinez-Hernandez and Assaf, 2023). In recent years, robotic grippers have been widely used in manufacturing processes to accelerate the processes and reduce labor costs (Cortinovis et al., 2023), (Georgopoulou et al., 2021). Nevertheless, the risk of object slippage provides significant challenges when manipulating manufacturing goods using robotic grippers in a robotic system. If the objects being handled accidentally slip out of the gripper, there is a considerable risk of damage, which could result in production delays and escalated costs. Therefore, early detection of object slip is essential (Yan et al., 2022).

A slip event in grasping is the unintentional movement that occurs between a grasped object and a robotic gripper while it is being manipulated. Slips are the result of insufficient frictional forces between the gripper and the object, which can result in a potential loss of control and diminished handling performance (Romeo and Zollo, 2020). Various methods utilizing tactile sensors have been established to mitigate object slippage in robotic gripper applications. An approach was presented for utilizing a three-fingered hand equipped with integrated optical tactile sensors to detect slip (James and Lepora, 2020). A novel tactile sensor, inspired by the structure of finger skin and based on optical microfiber, has been developed for slip detection (Jiang et al., 2021). These sensors offer accurate and rapid slip estimation. However, optical-based sensors require a significant amount of space for integration into robotic grippers. On the other hand, magnetic force sensors have been introduced to the application with robotic grippers to detect slips (Rosle et al., 2020; Le Signor et al., 2023). In principle, the slip causes a deformation in the elastic material embedded with magnets, which then changes the magnetic field. This change is detected by magnetic-based sensors and converted into electrical signals. However, magnetic-based sensors are not ideal to be utilized when dealing with metallic materials due to the potential for magnetic interference (Rosle et al., 2019). An elastomer-coated microelectromechanical (MEMS) barometric pressure sensor was introduced (De Clercq et al., 2022). However, the MEMS sensor requires high fabrication costs and tedious fabrication techniques.

The piezoelectric tactile sensor, which employs polyvinylidene fluoride (PVDF) to produce an electric charge in response to vibrations, demonstrates exceptional sensitivity to high frequencies. PVDF possesses exceptional characteristics, including outstanding resistance to chemicals, greater thermal stability, and low electrical permittivities (Xin et al., 2016; Wang et al., 2022). Such sensors have primarily been developed to detect vibrations in machinery and medical devices that require accurate vibration signals for optimal functioning. The piezoelectric sensor captures vibrations from the chest and converts them into an electrical output voltage signal that accurately represents respiratory and cardiac activities (Ji and Zhang, 2022). A PVDF film-based stretchable strain sensor that can detect joint movements and monitor vibrations in the environment without the need for external power was introduced (Guo et al., 2019). A sensor was designed as an accelerometer using a Graphene/PVDF nanocomposite to identify the subtle vibrations of industrial machines that occur at low frequencies and have low amplitudes (Maharjan et al., 2023). Nevertheless, such sensors require complex and tedious fabrication procedures, which results in higher fabrication costs.

In recent years, piezoelectric tactile sensors have been developed to detect vibrations by employing a sensing element that is directly attached to soft body layers. A tactile sensor array composed of PVDF films with soft PDMS films attached is designed to detect gentle slipping, touching, and bending for the purpose of monitoring human health is presented (Lin et al., 2021). In addition, piezo vibration elements are embedded in a soft fingertip made of silicone rubber for the purpose of texture roughness recognition using machine learning methods (Guo et al., 2024). However, to the best of the authors’ knowledge, the analysis on the effect of the thicknesses of soft body layers attached to sensing elements on the sensor’s performance to detect slip has not yet been addressed. It is expected that the vibrations are significantly influenced by the thickness of soft body layers, which in turn determines the piezoelectric tactile sensor’s performance to detect slip.

In this paper, a simple-structured and low cost of PVDF-based flexible piezoelectric tactile sensors to detect slip is presented. The effect of different thicknesses of soft body layer on the sensor’s performance to detect slip using robotic gripper is provided. The proposed sensor’s structure consists of a PVDF-based sensor, a soft layer, and a hard base. The fabricated sensors were attached to a motor-driven robotic gripper that was controlled by microcontroller. When the gripper holds onto an object and it slips, it causes vibrations in the soft body layer. The sensor will detect and interpret vibrations induced by soft body layer as slip events. The performance of the sensor was verified by slip test with different thicknesses of soft body layer Then, the slip test with different load of weight was performed to find the optimal thickness of the soft body layer for slip estimation using robotic gripper. In this brief report, the scope is limited to a soft body layer with a hardness of Shore A 30, and the grasped object is restricted to a plastic bottle, for repeatable forcing, avoiding confounding effects from widely varying surface frictions and textures. Furthermore, the flexible nature and mechanical adaptability of the sensor, which allows it to conform to various surfaces of contact objects, make it well-suited for use in manufacturing processes involving electric and electronic components, as well as for picking up and placing non-metallic objects.

## 2 Methodology

This section discusses the structure, principle, and the fabrication of the piezoelectric sensor. The fabrication requires simple steps and lower costs. The slip tests to validate the performance of the sensor are detailed in this section.

### 2.1 Design and fabrication of flexible PVDF-based piezoelectric tactile sensor

**FIGURE 1:**
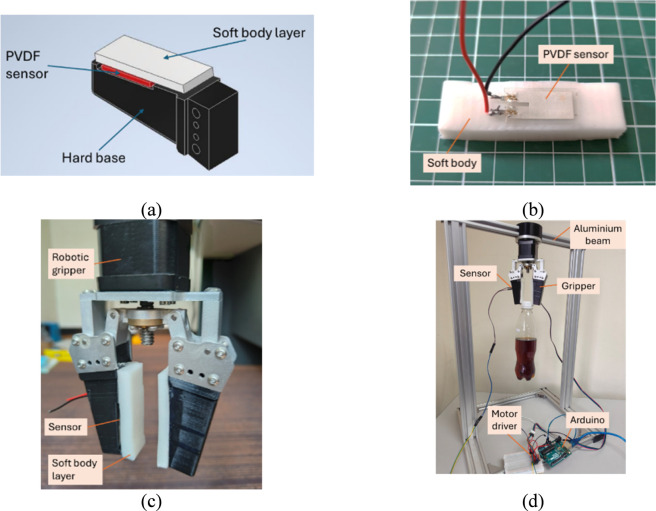
**(a)** Design of the PVDF-based piezoelectric tactile sensor. **(b)** PVDF sensor attached to a soft body layer. **(c)** Soft body layer with embedded PVDF sensor mounted on a robotic gripper. **(d)** Robotic gripper, connected to an Arduino and motor driver, gripping a plastic bottle at its cap.

Figure 1a shows the proposed design of flexible PVDF-based piezoelectric tactile sensor. The sensor consists of the soft body, the PVDF sensor, and the hard base. The soft body layer made from a silicon rubber with shore hardness of A30 was prepared by the casting process. The hard base that serves as a finger for robotic gripper was 3D printed. In this project, the PVDF sensor (LDT0-028K, TE Connectivity Co. Ltd.) that comprises a 28 µm thick piezoelectric PVDF polymer film laminated to a 0.125 mm polyester substrate was used. The PVDF sensor was attached to the soft body layer using adhesive, as shown in Figure 1b. Then, the soft body layer was attached to the hard base. In principle, when the piezoelectric film is positioned away from the mechanical neutral axis, it experiences higher strain, which consequently induces larger voltages within the piezopolymer. The soft and flexible properties of the soft body layer allow the sensor to adapt to the shape of the object being grasped, facilitating its ability to adjust to various shapes and surfaces of objects.

The PVDF sensor was connected to Arduino for output reading using analog signal communication. The proposed sensor was mounted onto one finger of a robotic gripper (12 V motor-driven two-fingered robotic gripper, RobotDigg Co. Ltd.), as shown in Figure 1c. The robotic gripper was suspended from a rigid aluminum bar for the experimental setup, as shown in Figure 1d. The object will be grasped by a robotic gripper equipped with the proposed sensor, and the soft body layer will deform its shape. When the object slips, the soft body layer will vibrate, which will be detected by the PVDF sensor as the thin piezo film deflects from its neutral axis, resulting in a signal output.

**FIGURE 2:**
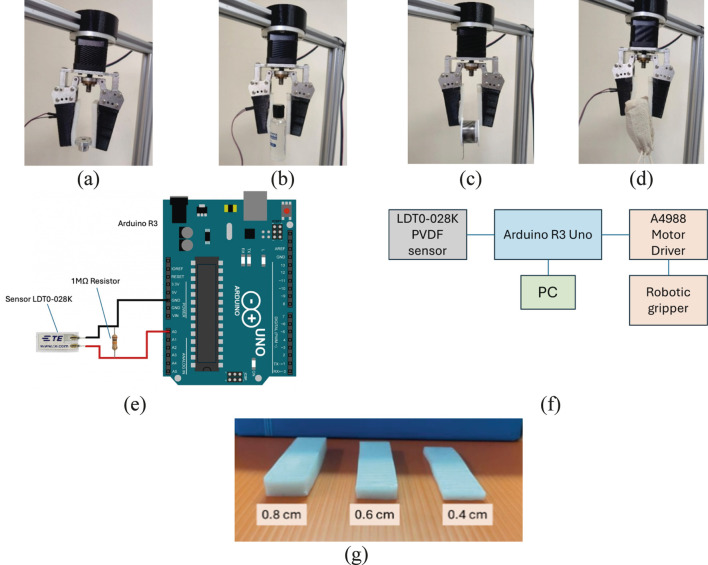
Robotic gripper embedded with the developed PVDF-based piezoelectric tactile sensor gripping various objects: **(a)** 95 g metal motor key hub, **(b)** 210 g small plastic bottle, **(c)** 160 g solder wire coil, and **(d)** 270 g small box covered with a cotton fabric bag. **(e)** The connection circuit diagram between Arduino R3 and sensor LDT0-028K. **(f)** Overall hardware connection diagram of the electrical devices. **(g)** Fabricated soft body layers with different thicknesses.

Figures 2a–d demonstrates the setup of the robotic gripper, which was equipped with the proposed flexible PVDF-based piezoelectric tactile sensor, to grip various kinds of objects including a metal motor key hub, a small plastic bottle, a solder wire coil, and a small box covered with a cotton fabric bag. The sensor was applied to one finger of the robotic gripper. As shown in Figure 2e, the PVDF sensor (LDT0-028K) is connected between the Arduino Uno R3 analog input (A0) and ground, with a 1 MΩ resistor placed in parallel as a load to discharge the sensor and convert the generated piezoelectric charge into a measurable voltage. In this work, the sensor output was sampled by the 10-bit ADC and streamed to the PC via USB at a baud rate of 19,200 bps, which is sufficient to capture the onset and evolution of slip events.

The DC motor controller (A4988 Driver, Allegro MicroSystems) was used to control the robotic gripper by adjusting the motor’s rotation and speed. Figure 2f illustrates the overall connection diagram of the electrical devices. The grasping test confirms that the robotic gripper, equipped with the proposed sensor, successfully grasped different types of object surfaces, including metal, plastic, PVC, and fabric. The soft body layer made contact with the object, and because of its mechanical flexibility, it changed its shape to securely grasp the object.

The natural frequency is the inherent oscillatory behavior of a system when it is disturbed from its equilibrium state and allowed to vibrate freely without any external forces acting upon it. The natural frequency is one of the characteristic properties of the system and is determined by its physical properties such as mass, stiffness, and geometry. In general, the natural frequency of a system, $\omega_{n}$ (rad/s), is determined by the stiffness and mass of the system. In a simple mass-spring system, natural frequency can be denoted as:

$$\omega_{n}=\sqrt{\frac{k}{m}}$$ (1)

where *k* and *m* are spring coefficient and mass of a system. In this project, the performance of the sensors is determined by natural frequency, i.e., the oscillatory behavior of the soft body layer during slip event of the grasped object. The natural frequency is proportional to the stiffness of the material, and inversely proportional to the mass. Therefore, the lower mass and the higher stiffness of the body layer is assumed to increase the natural frequency of the system, thus, increased the sensitivity of the proposed sensor. By managing the natural frequency of the soft body material, the sensor’s sensitivity can be determined.

In addition, the performance of the proposed sensor is also determined by the amplitude of the vibrations generated during the slip. Amplitude refers to the maximum displacement of the vibrating object, i.e., soft body layer from its equilibrium position. The equation of motion for a vibrating beam in the case of a vibrating plate or beam (Oliveto et al., 1997) can be derived from Euler-Bernoulli beam theory, as shown in Equation 2 below:

$$EI\frac{\partial^{4}\omega }{\partial x^{4}}+\rho A\frac{\partial^{2}\omega }{\partial t^{2}}=F\left(x,t\right)$$ (2)

The parameters of *E*, *I*, *ω*, *x*, *t*, *ρ*, *A*, and $F\left(x,t\right)$ represent the Young’s modulus, the area moment of inertia of the beam’s cross-section, the transverse displacement of the beam, the position along the length of the beam, time, density of the material, cross-sectional area of the beam, and the applied force per unit length, respectively. Assume the soft body layer is a beam shape. As discussed in (Loya et al., 2022), the natural frequency of the soft body, $f_{n}$ (Hz) layer can be approximately determined by:

$$f_{n}=\frac{\beta_{n}^{2}}{2\pi L^{2}}\sqrt{\frac{EI}{\rho A}}$$ (3)

where the parameters of $f_{n}$ , *L*, and $\beta_{n}$ depict the natural frequency, length of the beam, and a constant that depends on the mode shape and boundary conditions. The beam is assumed to be slender, homogeneous, and isotropic, and that plane sections remain normal to the beam’s axis both before and after bending. When a force is applied to the material, the amplitude of the resulting vibrations is related to the energy imparted by the force and the ability of the material to resist deformation.

Apart from natural frequency, the damping effect also plays an important role in determining the sensitivity of the sensor. Increasing the thickness of the soft layer enhances material damping, allowing more of the vibrational energy generated during a slip event to be absorbed and dissipated. As a result, the vibrations transmitted to the PVDF sensor are reduced, leading to a lower output signal. The relationship between natural frequency and damping is expressed through the damped oscillation frequency, $\omega_{d}$ :

$$\omega_{d}=\omega_{n}\sqrt{1-\zeta^{2}}$$ (4)

where *ζ* is the damping ratio. An increase in layer thickness leads to a higher damping ratio (*ζ*), which suppresses the vibration amplitude more significantly than it shifts the oscillation frequency. It can be demonstrated through impact damping tests that phenyl silicone rubber samples with increased thickness show lower acceleration amplitudes, confirming that thicker layers dissipate more energy and thus achieve a higher damping ratio (Yu et al., 2019). This is attributed to the material’s viscoelastic nature, where the internal friction of the polymer chains converts mechanical energy into heat. A thicker cross-section allows for more of this internal friction to occur as the material is cyclically stressed, thus dissipating more energy. Furthermore, through dynamic mechanical analysis and modal testing of a viscoelastic damping material (acrylic-based film on steel substrate), it was demonstrated that an increase in the thickness of the damping layer results in a higher damping ratio, confirming thickness as a key factor in improving vibration suppression (Wang et al., 2022).

### 2.2 Slip test with different thickness of soft body layer

The objective of the test is to investigate the performance of the proposed sensor with different thicknesses of soft body layer. In the test, as depicted in Figure 2g, three different thicknesses of soft body layer were fabricated, i.e., 0.8 cm, 0.6 cm, and 0.4 cm. The soft body layer were fabricated by casting processes using 3D printed mould. Each soft body layer was attached to a pair of finger of robotic gripper for test. During each grasping test, the robotic gripper consistently applied a force of 10 N. A plastic bottle was selected as the object to be held. The bottle was filled with water, and the total weight of the bottle and water was 350 g. Then, the robotic gripper firmly gripped at the bottle’s cap, as shown in Figure 1d. The bottle was slowly slipped out of the gripper due to its weight. The vibration of the body layer was generated due to the slip event of the plastic bottle, and the PVDF-based piezoelectric tactile sensor was used to capture the slip event by acquiring the sensor’s voltage signals. The slip test was repeated for three different thicknesses of soft body layer, and the sensor’s signal output was plotted in graph.

### 2.3 Slip test with different weights of objects

The aim of this test is to investigate the performance of the proposed PVDF-based piezoelectric tactile sensor in different weights of the grasped objects. In this test, the objects to be grasped by the robotic gripper were three different weights of water-filled plastic bottles, i.e., 250 g, 300 g, and 350 g. The soft body layer of 0.4 cm thickness was applied to the robotic gripper. Then, the bottle was gripped at the bottle’s cap, with the experimental setup shown in Figure 1d. The vibration of the soft body layer induced by the slip of the bottle due to gravity was recorded by the PVDF sensor’s signal output. The steps above were repeated with soft body layer thicknesses of 0.6 cm and 0.8 cm. The sensor’s output signal were recorded and plotted in graphs.

## 3 Results and discussions

### 3.1 Slip test with different thickness of soft body layer

**FIGURE 3:**
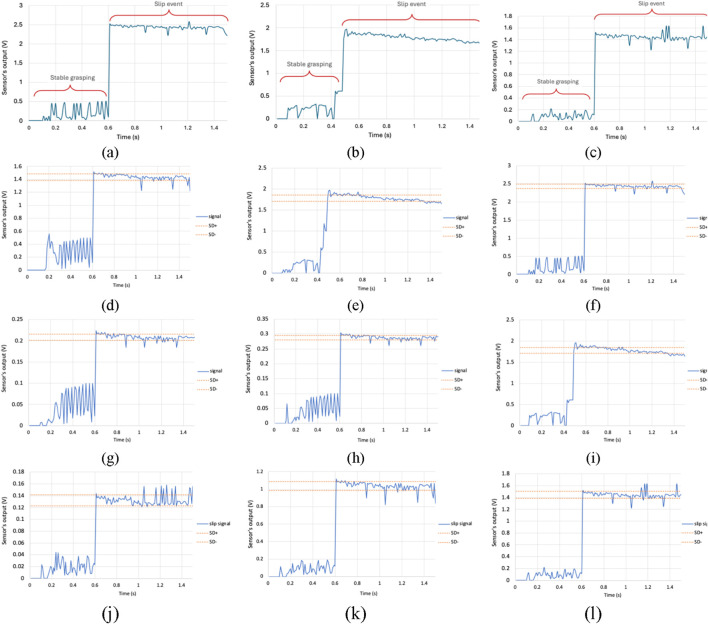
Sensor signals of the developed PVDF-based flexible piezoelectric tactile sensor. **(a–c)** Responses for different soft body layer thicknesses: **(a)** 0.4 cm, **(b)** 0.6 cm, and **(c)** 0.8 cm. **(d–l)** Responses during slip events for various combinations of layer thickness and object weight: **(d)** 0.4 cm, 250 g; **(e)** 0.4 cm, 300 g; **(f)** 0.4 cm, 350 g; **(g)** 0.6 cm, 250 g; **(h)** 0.6 cm, 300 g; **(i)** 0.6 cm, 350 g; **(j)** 0.8 cm, 250 g; **(k)** 0.8 cm, 300 g; and **(l)** 0.8 cm, 350 g.

The slip test results for soft body layers of thicknesses 0.4 cm, 0.6 cm, and 0.8 cm are given in Figures 3a–c, respectively. In Figure 3a, during gasping with no slip, the sensor’s signal output was stabilizing below 0.6 V. Then, when the bottle slipped, the output value of the sensor’s signal increased to a range of 2.2–2.6 V as a result of induced vibrations. In Figure 3b, the sensor’s signal output during stable grasping gave approximately below 0.8 V, and the signals consolidated between 1.6–2 V during slip event. The signal value stabilized below 0.3 V for 0.8 cm thickness and increased to between range of 1.2–1.7 V during slip, as shown in Figure 3c. These results demonstrate that higher layer thickness leads to lower sensor signal amplitudes during slip events. This behavior is attributed to the damping effect of thicker layers, which reduce vibration transmission under constant shear force. Consequently, lower thickness of soft body layers enhance sensitivity for slip detection.

It can therefore be concluded that a lower thickness of soft body layer yields higher sensitivity due to lower damping of vibration amplitudes. As expressed in Equation 3, a decrease in layer thickness reduces the moment of inertia (*I*), resulting in a softer structure that tends to lower the natural frequency. However, the reduction in thickness also decreases the mass per unit length (*ρA*), which in turn increases the natural frequency. Overall, the system becomes less massive and less damped, leading to a net increase in the natural frequency and a stronger vibrational response. In addition to this mass–stiffness effect, the damping effect also plays an important role in determining the sensitivity of the sensor. As described by the damped oscillation frequency in Equation 4, the damping ratio *ζ* influences the dynamic response of the system. An increase in layer thickness leads to a higher damping ratio (*ζ*), which suppresses the vibration amplitude more significantly than it shifts the oscillation frequency. Since a lower thickness of soft body layer has lower mass and a lower effective damping ratio, it absorbs and dissipates less of the energy generated during slip events, thus producing higher vibration amplitudes for a given force, as the vibrational energy is distributed over a smaller and less damped mass.

This study isolated the role of soft-body thickness at a fixed Shore A30 to avoid confounding with material stiffness. Theoretically, increasing hardness, i.e., higher elastic modulus E is expected to increase the layer’s modal frequency while also raising damping, leading to a trade-off between bandwidth and amplitude. To maintain consistency, the scope was therefore restricted to Shore A 30.

### 3.2 Slip test with different weights of objects

Figures 3d–l presents the results of slip test with different weights of objects. The standard deviation (SD) for each slip test is also presented in the figure. Figures 3g–i and Figures 3j–l show the sensor’s signal output with different weights (250 g, 300 g, and 350 g) of grasped objects for 0.6 cm and 0.8 cm of soft body layer, respectively. In all tests, the signal stabilized below a certain output value during stable grasping (no slip) and increased to a higher consolidated range of output values during slip events, reflecting vibration-induced responses to object slippage.

**FIGURE 4:**
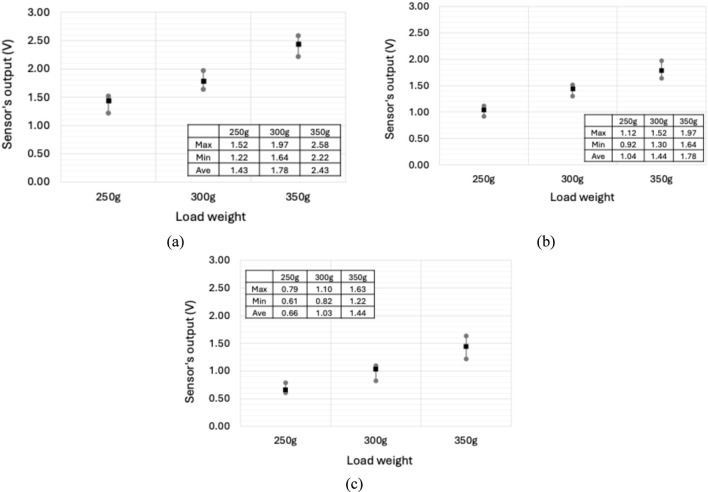
Average, minimum, and maximum sensor signal outputs during slip events for different soft body layer thicknesses: **(a)** 0.4 cm, **(b)** 0.6 cm, and **(c)** 0.8 cm.

The minimum, maximum, and average sensor signal outputs during slip events with different object weights for 0.4 cm, 0.6 cm, and 0.8 cm soft body layers are presented in Figures 4a–c, respectively. As shown in Figure 4a, the 0.4 cm layer produced the highest vibration response during slip, indicated by the higher sensor output, with a range of 1.22–2.58 V across slip trials with 250 g, 300 g, and 350 g object weights. In contrast, the 0.8 cm layer generated the lowest output signals, with a range of 0.61–1.63 V across all three slip test trials with different weights, as shown in Figure 4c. Higher vibration amplitudes during slip indicate greater sensor sensitivity. This is attributed to the lower mass of thinner soft body layers (*m*), which increases the natural frequency ( $\omega_{n}$ ) as described in Equation 1, making the system more prone to oscillations. Based on the minimum signal outputs observed during slip, the appropriate slip detection thresholds for the 0.4 cm, 0.6 cm, and 0.8 cm layers are estimated to be 0.8 V, 0.6 V, and 0.4 V, respectively. These suggested thresholds can be used to identify slip events during grasping, as they represent the minimum detectable slip-induced voltage change in the present configuration.

Moreover, the results in Figure 4 indicate that the average signal output decreases with a reduction in the weight of the grasped objects, as consistently observed in the slip tests conducted with different soft body layer thicknesses. When an object is grasped, the forces acting on it include gravitational force, inertial forces due to acceleration, and the normal and frictional forces applied by the gripper. During object slippage, the frictional force ( $F_{fric}$ ), the normal force (*N*), and the coefficient of friction between the object and the gripper (*μ*) can be expressed as in Equation 5:

$$F_{fric}=\mu N.$$ (5)

The normal force applied by the gripper (*N*) is defined as N=mg , where *m* is the mass of the object and *g* is the gravitational acceleration. Considering the overall proposed gripper system as a simplified mass–spring–damper model, the equation of motion can be expressed as in Equation 6:

$$m\ddot{x}+c\dot{x}+kx=F\left(t\right).$$ (6)

Therefore, heavier objects generate higher frictional forces to initiate slip. A larger friction force during slip results in a larger external force $F\left(t\right)$ in the equation of motion, which in turn leads to higher acceleration $\ddot{x}$ and velocity $\dot{x}$ of the mass, assuming the damping coefficient *c* and stiffness *k* of the system remain constant. The proposed sensor embedded in the gripper detects these vibrations, which are proportional to the acceleration and velocity of the mass. Furthermore, an increase in mass (heavier object) reduces the natural frequency of the system, as shown in Equation 1. A lower natural frequency leads to larger displacement amplitudes for a given stiffness, thereby contributing to higher vibration levels. In summary, heavier objects produce stronger vibrations during slip due to the combined effects of increased frictional forces and a lower natural frequency.

In steady state, the vibration amplitude is given by Equation 7:

$$\left|X\left(\omega \right)\right|=\frac{F_{o}/k}{\sqrt{{\left({1-\left(\omega /\omega_{n}\right)}^{2}\right)}^{2}+{\left(2\zeta \omega /\omega_{n}\right)}^{2}}}$$ (7)

where $\omega_{n}$ is the natural frequency and the damping ratio $\zeta =c/\left(2\sqrt{km}\right)$ . Since the PVDF film is bonded to the deforming soft layer, its output voltage magnitude can be expressed as $|V_{out}\left(\omega \right)|=K|X\left(\omega \right)|$ , with *K* is a proportionality constant determined by the PVDF piezoelectric properties, geometry, and electrical load. When the excitation frequency approaches the natural (resonant) frequency of the combined soft-body–PVDF structure, the deformation of the soft layer increases and the PVDF generates a larger voltage.

## 4 Conclusion

This paper presented the preliminary result of a flexible piezoelectric tactile sensor based on PVDF for slip detection in a robotic gripper. The sensor structure consists of a PVDF film attached to a soft rectangular body layer, mounted on a rigid base acting as the gripper. During slip events, vibrations generated in the soft body layer displace the piezo film from the mechanical neutral axis, producing measurable voltage outputs. Thus, slip can be identified through variations in the sensor’s output signal. This study investigates the influence of soft-cover thickness on PVDF-based slip sensing under controlled conditions. Hardness (Shore A 30), geometry, and grasp pose were held constant, and a minimal object set was employed to ensure repeatable forcing while avoiding confounding effects from variations in surface friction and texture. Grasping tests with different objects demonstrated that the proposed gripper, embedded with the developed sensors, can reliably grasp objects with varying surface properties. Slip tests under different body layer thicknesses and object weights further showed that thinner soft body layers enhance sensitivity, as the reduced mass of the system results in higher vibration amplitudes. Furthermore, lighter objects produced lower signal outputs, attributed to reduced frictional forces and higher natural frequencies, which generate lower vibrations during slip. Overall, the results highlight the feasibility of employing flexible PVDF-based tactile sensors for slip estimation in robotic grippers. Qualitative grasps on metal, plastic, PVC coil, and fabric-covered items were also included to demonstrate face validity across distinct surface types. Comprehensive catalog-level benchmarking is valuable for production deployment and is identified as future work, after finalizing the design rule for cover thickness. Detailed calibration to map the output voltage to absolute normal force or acceleration across frequency will be addressed in future work. Future work will also focus on integrating multiple PVDF sensors and employing machine learning techniques to improve slip detection accuracy and robustness.

## Data Availability

The raw data supporting the conclusions of this article will be made available by the authors, without undue reservation.
